# PhenoMATRIX™ for the screening of Group B *Streptococcus* (GBS) carriage in pregnant women: ready to get rid of the LIM broth?

**DOI:** 10.1007/s10096-024-04985-7

**Published:** 2024-11-09

**Authors:** Abdessalam Cherkaoui, Gesuele Renzi, Jacques Schrenzel

**Affiliations:** 1 Bacteriology Laboratory, Division of Laboratory Medicine, Department of Diagnostics, Geneva University Hospitals, 4 Rue Gabrielle-Perret-Gentil, 1205 Geneva, Switzerland; 2 Genomic Research Laboratory, Division of Infectious Diseases, Department of Medicine, Geneva University Hospitals and Faculty of Medicine, Geneva, Switzerland

**Keywords:** Group B *Streptococcus*, GBS, *Streptococcus agalactiae*, Chromogenic media, WASPLab, PhenoMATRIX, LIM broth

## Abstract

There is a constant need to reduce turn-around times and keep costs as low as possible for the carriage screening of GBS in pregnant patients. Laboratory automation might provide an edge in this field. The objectives of the present study were: i) to compare the performance of the direct chromID™ Strepto B agar (CA) plating against LIM-broth enriched plating on CA for the detection of GBS from vagino-rectal screening-swabs; and ii) to assess the usage of PhenoMATRIX™ for the automated screening of GBS. Between January 2021 and December 2023, 9′107 vagino-rectal specimens were collected from pregnant women at Geneva University Hospitals and were used to address the first objective. There was a small difference in the GBS detection rates between direct CA plating (13.2%; 1′202/9′107) and LIM-broth enriched plating on CA (13.2%; 1′198/9′107). Based on the LIM-broth enrichment results, the sensitivity and specificity of the direct CA plating were 98.3% (95% CI, 97.3%—98.9%) and 99.7% (95% CI, 99.5%—99.8%), respectively. Importantly, for 25 specimens, GBS growth was only detected by direct CA plating. We used a random set of 8′768 CA plate pictures for the machine learning of PhenoMATRIX™. The validation was carried out on an additional set of 830 CA plate pictures. The sensitivity and specificity of PhenoMATRIX™ were 100% (95% CI, 96.6%—100.0%) and 90.2% (95% CI, 87.8%—92.1%), respectively. We established that for GBS screening, the performance of direct CA plating is not inferior to the LIM-broth enriched approach. By relying on PhenoMATRIX™, the negative predictive value for GBS screening reaches 100% (95% CI, 99.4%—100.0%), enabling the automatic release of GBS-negative cases within 24 h.

## Introduction

Group B *Streptococcus* (GBS or *Streptococcus agalactiae*) is an encapsulated Gram-positive commensal bacterium that frequently colonizes the gastrointestinal and genital tracts. While GBS is typically harmless, it remains the most common cause of early-onset neonatal infections including pneumonia, meningitis, and sepsis in infants [1, 2]. The elderly and immunocompromised persons are also a population at risk yet presenting with more diverse clinical presentations that typically require rapid and comprehensive medical care [3–5]. The percentage of GBS-colonized pregnant women amounts up to 40% [6]. In preterm infants the mortality rate of early-onset GBS disease can reach up to 25% [7, 8], whereas it is much lower (< 3%) in term infants [8, 9]. The mother-to-infant transmission happens, usually, when GBS reaches the amniotic fluid after the rupture of the membranes. GBS are also capable to cross intact membranes through transcytosis. The other common possibilities of contamination occur during the transit through the birth canal [10]. Various culture guidelines have been issued for the prevention of perinatal GBS disease. They typically recommend the use of a selective enriched broth culture from vagino-rectal screening swabs performed during the late stage of pregnancy. Intrapartum antibiotic prophylaxis is then recommended for GBS-colonized pregnant women, according to identified risk factors [9, 11–13].

Various diagnostic approaches such as LAMP, qPCR, or biosensors are currently available [14–17]. The molecular assays are more time efficient especially without pre-enrichment, but they remain relatively expensive for routine GBS screening. Hence, they are typically applied in only specific cases, according to defined risk factors. The opportunity to further reduce turn-around times while keeping costs as low as possible might be tackled by laboratory automation.

LIM broth enrichment with subsequent subculture on blood agar was widely recognized as the gold standard method for routine vagino-rectal screening of GBS. Currently, U.S. and most of European screening-based guidelines advice Columbia CNA agar or chromogenic agar instead of blood agar plates [18, 19]. Cultivation methods lack sensitivity as compared to molecular assays and provide delayed reporting with results requiring at least 48 h. However, culture has recently benefited from the implementation of total laboratory automation (TLA) that has in turn significantly reduced the incubation times, enabling earlier plate culture readings [20–22]. In this sense, the management of GBS screening might be significantly enhanced using TLA coupled to artificial intelligence (AI). The WASPLab™ (Copan Diagnostics) is one of the TLA systems thoroughly assessed and validated. The WASPLab™ includes an up-front specimen processing module (WASP™), an integrated track lines, and incubators integrating a digital imaging system to record images of plates at pre-defined time points. PhenoMATRIX™ software (Copan) is an AI module enabling the development of accurate specific algorithms to evaluate and sort culture media plates based on the digital plate images, as evaluated in our previous study [23].

The objectives of the present study were: i) to compare the performance of the direct chromID™ Strepto B agar (CA) plating using WASPLab against LIM-broth enriched plating on CA for the detection of GBS from vagino-rectal screening-ESwabs; and ii) to assess the usage of PhenoMATRIX™ for the automated screening of GBS.

## Sample processing and manual work-up

Between January 2021 and December 2023, 9′107 vagino-rectal swab specimens (ESwab, Copan Diagnostics, Brescia, Italy) were collected from pregnant women at Geneva University Hospitals (HUG). One nylon flocked swab was used for sampling the patient and then submerged into 1 ml of ESwab transport medium (Copan). The samples were sent to the bacteriology laboratory for GBS culture. Direct plating is carried out using the WASP by inoculating 30 µl from the ESwab transport medium onto chromID™ Strepto B agar (CA) (BioMérieux, Marcy l’Etoile, France) and another 30 µl into a LIM-enrichment broth™ (Copan). The CA is incubated for 24 h in the WASPLab™ under aerobic condition. The LIM broth is incubated overnight at 37 °C using a traditional incubator and subcultured the following morning onto a CA plate, which is in turn incubated at 37 °C for 18 h using the WASPLab™. For each incubation time on the WASPLab™ (i.e., 0 h, 18 h, and 24 h), high-resolution digital images are taken according to the manufacturer‘s settings. The CA images are assessed for pale pink to red round and pearly colonies. The isolates are then confirmed as *S. agalactiae* by matrix-assisted laser desorption ionization–time of flight mass spectrometry (MALDI-TOF/MS) (MBT Compass 4.1, library version 11.0 (11′410 spectra), Bruker Daltonics, Bremen, Germany) according to the manufacturer’s instructions [24]. To guarantee maximal consistency, the direct CA plating results were kept blind from the results provided by the LIM-enrichment broth subcultures on CA. All plate images were read manually by the lab technologists.

The positive GBS cultures were semi-quantitatively evaluated according to a 4 points arbitrary scale of 1 + , 2 + , 3 + or 4 + (scant, light, moderate or heavy), where moderate and heavy correspond to large GBS colony counts (i.e., > 100′000 per ml).

## GBS PhenoMATRIX™: training phase

We collected culture images of 8′768 CA media used for direct plating and the subculture of the LIM broth. All images were previously classified by manual work-up as either GBS-positive or -negative. These images were utilized for the machine learning approach (i.e., ground truth to train the convolutional neural networks).

## GBS PhenoMATRIX™: validation phase

The validation phase was carried out on another 830 CA, which corresponded to 437 non-duplicate vagino-rectal ESwab specimens sequentially referred to our laboratory plus 393 additional CA from the LIM broth subcultures. Digital images of the CA were prospectively analyzed by PhenoMATRIX™. To guarantee better consistency, the same CA images were read by the technologists blinded of the results returned by PhenoMATRIX**™**. All the discrepant results (n = 71) were confirmed by clinical microbiologists by reassessing the digital images (i.e., culture reading).

## Results

### Direct CA plating performance against LIM-broth enriched plating on CA

**Fig. 1:**
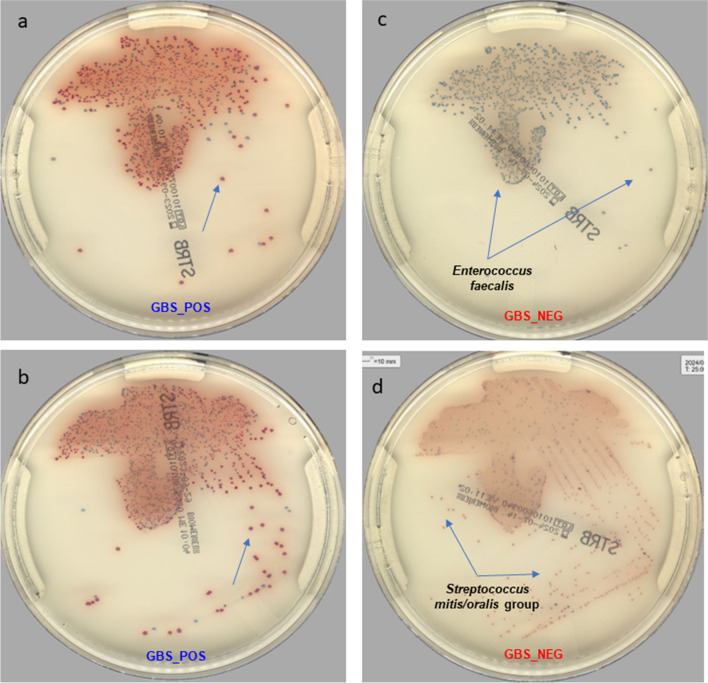
Representative digital pictures of the ChromID™ Strepto B agar growing GBS (**a** and **b**) and mixed flora (**c** and **d**)

Overall, 13.4% (1′223/9′107) of the vagino-rectal samples tested positive for GBS (Table 1). Among the 1′202 positive GBS detected by direct CA plating, 30.4% (366/1′202) were positive with 1 + of GBS, 19.8% (239/1′202) were positive with 2 + , and 49.8% (597/1′202) were positive with 3 + or 4 + (Figure 1a and 1b).

**Table 1:** Performance of direct CA plating against LIM Enrichment broth subcultured on CA for the screening of GBS from vagino-rectal ESwabs

		LIM Enrichment broth subcultured on CA				
		**Negative**	**Positive**	**Total**		
**Direct CA plating**	**Negative**	7884	21	**7905**	**Sensitivity**	98.3 (95% CI, 97.3%—98.9%)
	**Positive**	25	1177	**1202**	**Specificity**	99.7 (95% CI, 99.5%—99.8%)
	**Total**	**7909**	**1198**	**9107**	**Positive Predictive Value**	97.9 (95% CI, 96.9%—98.6%)
					**Negative Predictive Value**	99.7 (95% CI, 99.6%—99.8%)

*CA*, chromID™ Strepto B agar

Figure 1c and 1d show digital pictures of the ChromID™ Strepto B agar growing mixed flora. *Enterococcus* spp, *Lactobacillus* spp, and alpha-hemolytic *Streptococci* constituted the more important organisms identified on CA plates that could interfere with the GBS growth.

There was a small difference in the GBS detection rates between direct CA plating (13.2%; 1′202/9′107) and LIM enrichment broth plated on CA (13.2%; 1′198/9′107). According to the LIM broth enrichment results, the sensitivity and the specificity of the direct CA plating were 98.3% (95% confidence interval (CI), 97.3%—98.9%) and 99.7% (95% CI, 99.5%—99.8%), respectively (Table 1). Hence, the negative and the positive predictive values were 99.7% (95% CI, 99.6%—99.8%) and 97.9% (95% CI, 96.9%—98.6%), respectively.

**Fig. 2:**
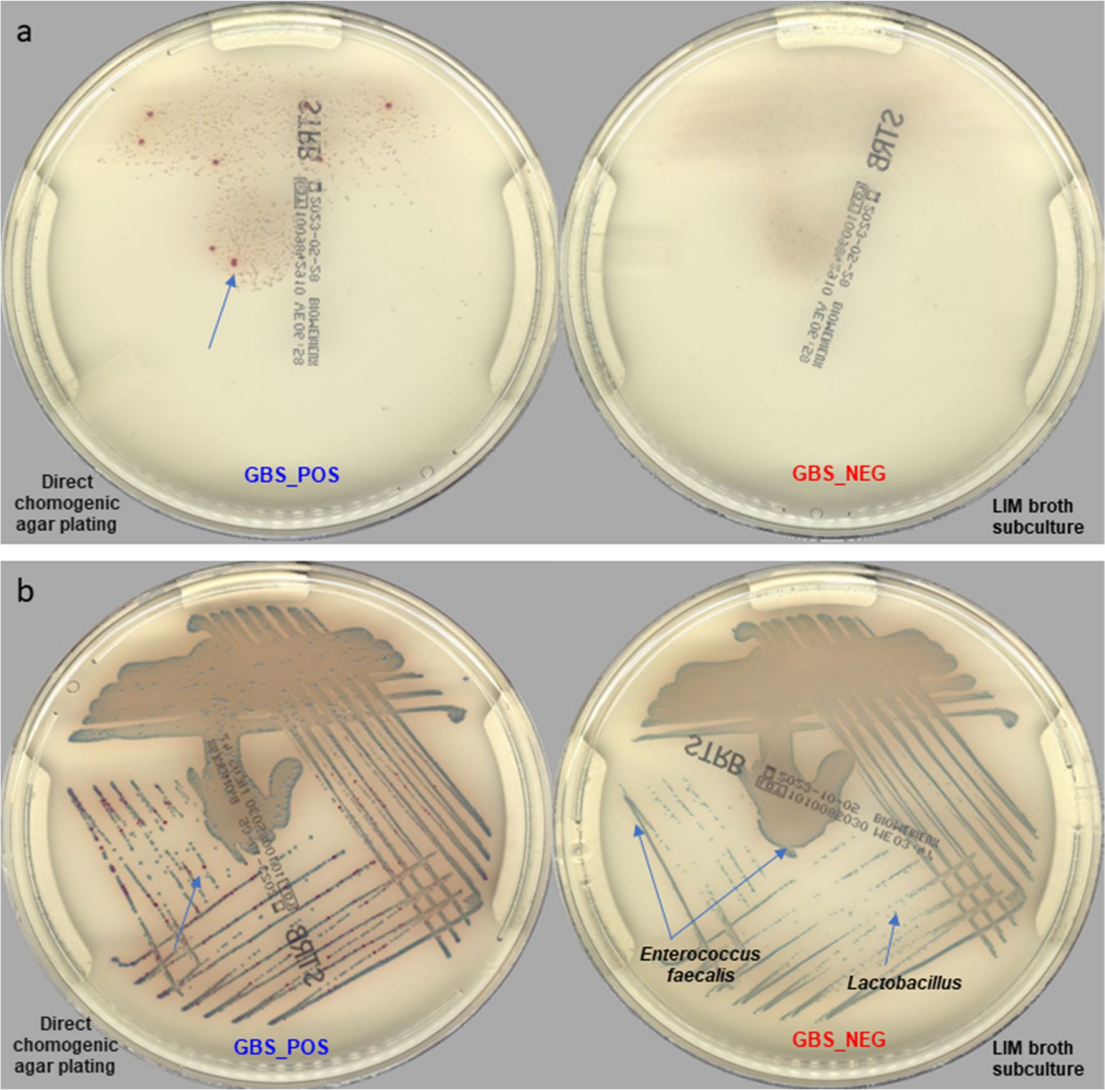
Digital pictures of the two LIM Enrichment broth subcultured on CA and leading to false negative results

When considering the 25 GBS-positive specimens on direct CA plating that were missed after LIM enrichment broth, the GBS inocula were in most cases (76%, 19/25) of low (1 +) abundance as only a few colonies were observed on the first CA media plate (Figure 2a). In the remaining cases (24%, 6/25), GBS inocula were light to heavy (2 + to 3 +) (Figure 2b). LIM-broth enriched plating on CA enabled the detection of an additional 21 positive cases that were missed by the direct CA plating.

### PhenoMATRIX™ performance for the detection of GBS

Overall, 830 CA media were analyzed by PhenoMATRIX**™** in comparison with the routine manual reading. This corresponded to 437 direct CA plating and 393 CA from the LIM broth subculture. The sensitivity and the specificity of PhenoMATRIX**™** for the direct CA plating were 100% (95% CI, 95.9%—100.0%) and 88.2% (95% CI, 84.3%—91.1%), respectively (Table 2a). For the 393 LIM broth plated on CA, the sensitivity, and the specificity of PhenoMATRIX™ were 100% (95% CI, 82.4%—100.0%) and 92.0% (95% CI, 88.8%—94.3%), respectively (Table 2b). Among the 830 CA media analyzed by PhenoMATRIX**™,** 71 were classified as false positive based on manual reading results. All false positive cases were reviewed by clinical microbiologists by reassessing the digital images, and the absence of GBS was established. Hence, the sensitivity and the specificity of PhenoMATRIX™ for the 830 CA media included in this study were 100% (95% CI, 96.6.4%—100.0%) and 90.2% (95% CI, 87.8%—92.1%), respectively (Table 2c). The negative and positive predictive values were therefore 100.0% (95% CI, 99.4%—100.0%) and 60.3% (95% CI, 54.9%—65.4%), respectively.

**Table 2:** Performance of PhenoMATRIX**™** for the detection of GBS from vagino-rectal screening-ESwabs using CA as compared to manual reading

		Manual work-up				
		Negative	Positive	Total		
PhenoMatrix^TM^ (Direct CA plating)	Negative	305	0	305	Sensitivity	100% (95% CI, 95.9%—100%)
	Positive	41	91	132	Specificity	88.2% (95% CI, 84.3%–91.1%)
	Total	346	91	437	Positive Predictive Value	55.8 (95% CI, 48.6%—62.7%)
					Negative Predictive Value	100 (95% CI, 98.8%—100%)
		Manual work-up				
		Negative	Positive	Total		
PhenoMatrix^TM^ (LIM-broth enriched plating on CA)	Negative	345	0	345	Sensitivity	100% (95% CI, 82.4%—100%)
	Positive	30	18	48	Specificity	92.0% (95% CI, 88.8%—94.3%)
	Total	375	18	393	Positive Predictive Value	65.1 (95% CI, 57%—72.5%)
					Negative Predictive Value	100 (95% CI, 98.9%—100%)
		Manual work-up				
		Negative	Positive	Total		
PhenoMatrix^TM^ (Direct CA plating and LIM-broth enriched plating on CA)	Negative	650	0	650	Sensitivity	100% (95% CI, 96.6% - 100%)
	Positive	71	109	180	Specificity	90.2% (95% CI, 87.8% - 92.1%)
	Total	721	109	830	Positive Predictive Value	60.3% (95% CI, 54.9% - 65.4%)
					Negative Predictive Value	100 (95% CI, 99.4% - 100%)

*CA* chromID™ Strepto B agar

## Discussion

### Direct CA plating versus LIM enrichment broth subcultured on CA

Over the last fifteen years, a wide range of studies were undertaken to resolve the particularly sensitive and complex question on the need -or not- to integrate a selective broth enrichment subculture for the screening of GBS in pregnant women. The large number of different solid culture media (chromogenic and non-chromogenic) as well as liquid culture media evaluated, the different specimen types (vaginal, rectal, or vagino-rectal) assessed, and the highly diverse protocols used may explain the heterogeneity of the data published [9, 19, 25, 26]. In the study conducted by Kwatra et al., [27] the authors compared the performance of the Todd-Hewitt broth with 8 µg/ml gentamicin and 15 µg/ml nalidixic acid (Trans-Vag broth) subcultured on 5% sheep blood agar (SBA) against the direct plating on chromID™ Strepto B agar (CA) or Columbia colistin-nalidixic agar (CNA) for the detection of GBS. For the 130 rectal swabs analyzed, the sensitivities of CA, CNA, and Trans-Vag broth subcultured on SBA were 88.4%, 72.1%, and 27.9%, respectively. A potential limitation of this study was the use of a non-selective medium for the subculture of the enrichment broth, which probably explains its poor performance for the detection of GBS from rectal swabs. In another study, El Aila et al., [28] found that the sensitivities of the six different GBS culture methods used for 300 swabs included in their study, namely direct plating onto CNA, GBSDA (Granada Medium), CA, LIM broth subcultured on CNA, LIM broth subcultured on GBSDA, and LIM broth subcultured on CA, were 56%, 95%, 100%, 78%, 96%, and 100%, respectively. The specificities were 84%, 100%, 96%, 84%, 100%, and 96%, respectively. In our study, the sensitivity and the specificity of direct CA plating were comparable to that of the LIM broth subcultured on CA, whereby the latter enabled the detection of only 1.7% (21/1′223) additional GBS positive samples. Even more important, LIM enrichment broth subcultured on CA missed 2.0% (25/1′223) of the GBS positive samples. In agreement with the studies mentioned above, our results establish that the performance of the direct CA plating is at least comparable with the LIM broth subcultures on CA. Moreover, this protocol can significantly reduce the turnaround time of the GBS screening. The cost-effectiveness of the direct CA plating is further increased by automating the cultures using WASPLab™ and saving reagents by omitting the broth enrichment phase, which in turn also contributes to reduce the workload. Finally, the detection limit of GBS using direct CA plating depends largely on the composition and abundance of the flora (e.g., *Enterococcus* spp, and alpha-hemolytic Streptococci) competing and masking the growth of GBS when the GBS inoculum is low.

### Detection of GBS from ChromID™ Strepto B agar using PhenoMATRIX™

The present study outlines the performance of PhenoMATRIX™ coupled to CA plate for the detection of GBS in routine screening swabs as compared to the routine manual work-up. The sensitivity and the specificity of PhenoMATRIX™ for the 830 CA plates analyzed in the present study were 100% (95% CI, 96.6.4%—100.0%) and 90.2% (95% CI, 87.8%—92.1%), respectively. The sensitivity and the specificity of PhenoMATRIX™ coupled to CA at 48 h according to the study performed by Baker et al. on 676 vagino-rectal swabs were 95.5% (95% CI, 90.5%—98.0%) and 63.0% (95% CI, 58.7%—67.2%), respectively [29]. In the study performed by Foschi et al. on 1′068 vagino-rectal swabs PhenoMATRIX™ revealed a sensitivity of 100% and a specificity of 64.5% [30]. As noted in our study, the specificity of the PhenoMATRIX™ algorithm was greatly improved by the manufacturer as compared to the previous settings. The false-positive results have been largely assigned to the moderate specificity of the chromogenic agar. Mixed overgrowth of various species of *Streptococcus* spp (*S. anginosus, S. mitis/oralis, S. parasanguinis, S. salivarius*), *Enterococcus* spp, and *Lactobacillus* spp. represented the most frequent false positive cases. In the present study the rate of false positivity remained moderate (8.6%, 71/830).

## Conclusions

In agreement with previous studies, we established that the performance of the direct CA plating is not inferior to the LIM enrichment broth subcultured on CA. Accordingly, the addition of the enrichment broth for GBS screening is not relevant in our setting.

Regarding PhenoMATRIX™, it is important to underline the very accurate identification of the negative GBS culture plates. The high negative predictive value enables the implementation of the automatic release of GBS-negative results, the automatic discharge of these negative plates from the incubators and their tossing using PhenoMATRIX™ PLUS, offering shorter turn-around times at low unitary cost.

## Data Availability

No datasets were generated or analysed during the current study.
